# The Problem of the Task. Pseudo-Interactivity as an Experimental Paradigm of Phenomenological Psychology

**DOI:** 10.3389/fpsyg.2020.00855

**Published:** 2020-04-30

**Authors:** Alexander Nicolai Wendt

**Affiliations:** Department of Psychology, Heidelberg University, Heidelberg, Germany

**Keywords:** phenomenological psychology, problem-solving, semantic complexity, pseudo-interactivity, psychology of thought

## Abstract

Real-life problems are almost always socially complex, even when we are by ourselves. Psychological problem-solving research must therefore integrate complexity as a domain of investigation. However, the simulation of complex interactions represents a major challenge to designing experiments dealing with the nature of social interaction: Simulated social interaction, even when enacted by confederates, is not identical to the actual social interaction. Subjects will tend to enact simulated interaction in distinct ways. To understand these differences, the different situation enactments ought to be analyzed psychologically. Essentially, an instruction to perform in an experimental setting cannot guarantee that the experimental subject will take a certain attitude toward the situation. Early psychology of thought considered the social nature of the experimental situation when discussing the notion of the task. Modern experimental psychology can draw on these reflections in order to grasp better the essential characteristics of social complexity and to establish pseudo-interactivity as a phenomenologically enriched experimental paradigm. Its methodological power is illustrated by an exploratory experimentation on problem-solving.

## Introduction

In the last century, the psychology of thought has partly developed into the psychology of problem-solving (for a historical and sociological overview see Kusch, 1999). In the beginning of this development, approaches like *Denkpsychologie* from Würzburg investigated the functions of higher cognition beyond associationism. Scholars, such as Karl Marbe, August Messer and, most importantly, Karl Bühler, proposed psychology of thought to be a field of research that dealt with the specifics of conscious experience in response to tasks: easy or complex. Thus, the notion of “task” became fundamental for their investigations.

The investigation of problem-solving is the externalist heritage of thought-psychology, mainly drawing on the notion of the task as it has been used, for example, by the De Groot (1946) in his seminal work on the psychology of chess, “Het denken van de schaker.” In his conceptual reflections, de Groot tends to use the terms of “task” and “problem” interchangeably. It is no wonder that Newell and Simon (1972), who had read de Groot’s thesis on chess before writing “Human Problem Solving,” also do not make a clear distinction between “task” and “problem.” Their groundbreaking *opus magnum* inspired all the contemporary research on problem-solving, implicitly bridging it with psychology of thought.

Unlike psychology of thought, recent investigations no longer consider non-inductive explanations. The original thought psychology, on the other hand, received its original inspiration by alternative epistemological approaches: Oswald Külpe, the founder of the *Würzburgian* Institute of Psychology and the father of psychology of thought, stated in his historical retrospection on the movement: “In epistemology, it is the problem of reality that came thought-psychology’s way. Already before the experimental investigation of thought, it had been remarked, especially by Twardowski, Husserl, Freytag, that the content of thought and its object are different and that it is not directed at itself but at something transcendent beyond its own sphere” (Külpe, 1922, p. 320; translation by the author).

It is a recurrent motif in the initial psychology of thought literature to refer to Husserl (see Münch, 1998), namely in Marbe (1901), Messer (1906), and Bühler (1907), but also in Selz (see Seebohm, 1970) and Lindworsky (1916). Thus, there may be a developmental trajectory of the phenomenological part of thought psychology that is similar to the psychological development via de Groot to Newell and Simon. But this is not only a historical alternative. Problem-solving research seems to have encountered an impasse, if not a deadlock (as it has been described by, e.g., Getzels, 1982; Quesada et al., 2005; Ohlsson, 2012; Funke, 2014; Wendt, 2018). Thus, the question must be posed whether the contemporary limitations of cognitivist problem-solving research result from the neglect of the epistemological problems that were apparent in the days of thought-psychology’s foundation.

To give an example, problem-solving research frequently presupposes the general motivational directedness of the experimental subjects toward the instructed situation, the so-called “properly motivated subject” (Newell and Simon, 1972, p. 54). Wertheimer labeled this presupposition as “constructed foundations” (for a discussion see Nerney, 1979). In other words, the researchers simply assume that giving an instruction means that the subjects will have the problem. Put more appropriately (and honestly), problem-solving research is not concerned with whether or not its subjects really have a problem in the authentic sense of the word or simply pass their time in the laboratory. It is a methodological concern if this difference can be neglected since it cannot be measured with precision, i.e., as a behavioral variable. Unlike contemporary cognitivists, the original psychologists of thought have at least discussed this question.

The original conundrum can be summarized as follows: Does it require theoretical and epistemological considerations in order to advance problem-solving research as an instance of behavioral cognitivism in psychology? Without making the case for any, merely ideological, response that advocates either empiricism or rationalism, it should be conceded that the way to an answer must directly deal with the psychological phenomena themselves. This approach demands a minimal adjustment to the inductive methodology of experimental psychology. This step may be called a phenomenological parallax. It does not entail a rigid rationalism, even though most radical empiricists may think so. Its basic contention is simply that the scientific concept formation is not self-given. Therefore, it is necessary to investigate the foundation of scientific concepts by intuition and reflection.

In the case of problem-solving research, this means that the experiential foundations of notions like “task” or even “problem” themselves (see Wendt, 2018) should be restored in order to facilitate both theoretical debate and theory-directed empirical investigation. It is not enough to rely on empiricist paradigms just because they give supposedly reliable or effective results. Their phenomenal relevance should be discussed if not proven beyond empirical traditions. Only this emancipation from any irrelevant experimental approaches will allow psychology to be a rigorous science and to establish criteria of quality that surpass the statistical control of internal consistency.

### What Is a Task?

From an externalist point of view, it might seem as though a task and, not differently, an experimental task could be adequately described as a material constellation. For example, Philipp and Koch claim that “the term task can be basically understood as ‘what subjects have to do in an experiment.”’ (Philipp and Koch, 2010, 383). This common conception presumes a constellation of the experimental subject and a goal state: “the link between a task and a goal is that a task can be assigned by a third party. […] It is then the duty of every single person to decide whether he or she accepts the task assignment. If he or she does, the depersonalized task becomes a personal goal of that specific person” (Künzell et al., 2018, p. 6).

Quite obviously, the notion of the goal is no less ambiguous than the notion of the task. Taken within an externalist framework, it entails both the instruction, i.e., an imperative communication by the examiner, and its representation: “the instructions given to subjects in an experiment must define the task(s) at a level that permits comprehension of what has to be accomplished” (Schneider and Logan, 2014, p. 29). Ultimately, the priority of this external input, this stimulation or stimulus of the instruction, is the onset and condition for any representational information processing, that is, problem-solving (in the respective sense).

Consequently, an externalist framework must attribute all variation in the motivation of subjects to differences in their representations of the same instruction. This explanatory approach is artificial since it overrates the separation between representations of the instruction and motivational processes that do not relate to the instruction. Furthermore, the construction of an instruction may make it empirically impossible that all subjects experience the same goal-directedness.

Most importantly, however, the externalist explanation cannot provide a sufficient understanding of the motivational dynamics of tasks: assuming that a task conveys an instruction via communication to a goal, the question remains why a person would want to allow this conveyance. In fact, this gap could even be understood as a second task, namely, the task to accept the experimental task. This explanatory insufficiency results in an infinite regress because the externalist notion of the task only presupposes the actual transformative faculty of the task. This is something that cannot be grasped from an externalist standpoint.

Psychology of thought, on the other hand, did not overlook the complexity of the conscious experiences and processes that underlie the experimental situation. Three members of the movement made important and successively more complex contributions to the understanding of these particular dynamics: Henry J. Watt in his “Experimental contributions to a theory of thought” from 1904, Narziß Ach’s “On the agency of volition and on thought” from 1905, and Otto Selz’ “On the laws of the orderly course of thought” from 1913. Bearing major methodological resemblance, the three texts are dedicated to the investigation of thought in its immanent psychological nature. Thus, the notion of the task is not only relevant as an experimental condition but in its psychological function.

### Henry J. Watt’s Observation of the Task

Watt’s approach was the critical evaluation of associationist explanations of thought. Associationist psychologists, like Georg Elias Müller or Theodor Ziehen (see Müller, 1913), had claimed that the responses to a given stimulus word could be explained exclusively by local associative links in memory. These links obey the associative laws in the tradition of Alexander Bain, David Hume and, ultimately, Aristotle. In other words, the traditional claim was that reproductions (sc. associations) result entirely from the strength of associative links that were given by a learning experience or disposition.

In his experiments, Watt revised this assumption under the condition of restricted, instead of free, association. At this point, it is essential to highlight the associationist conviction that there is an innate mechanism to respond to a stimulus. In this sense, the traditional notion of the task was trivial: When one sees a word or any other stimulus, they cannot but produce a reaction. Although it might not be entirely unfounded to assume humans, or even life itself, have a universal responsive nature, this mechanism cannot account for the specificity of experimental tasks because it is necessarily unrestricted, or, as Watt would say, free. This difference is reflected in the distinction between free and restricted associations. Likewise, it reflects the fundamental difference between the associative response mechanisms and tasks in the proper sense.

It becomes clear that, for Watt, a mere stimulus itself does not constitute a task – it is solely a free association^1^. Restricted associations become possible when the stimulus word is accompanied by a task in the specific sense of the word, such as finding “a superordinate concept,” “a subordinate concept,” “a whole,” “a part,” etc. Watt, then, observed that the variance of experimental responses almost entirely depended on the restriction given by the task, overriding the unspecific effect of the free association. His results show, for example, that the tasks of finding “a whole” or “a part” invite significantly more imaginative thoughts than the tasks of finding “a superordinate concept” or “a subordinate concept”. In contrast, in their introspective reports, the subjects reported more verbal experiences for these latter tasks.

An example can illustrate this observation: Given the stimulus “apple,” a subject is more likely to have a verbal association of the word “fruit” given the task “superordinate concept” but given the task “a whole,” the imaginative, i.e., visually imaginative, association of a tree becomes more likely. These results might not come as a surprise, but they contradict the traditional contention that the stimulus word’s associative links account for the responsive variance. Even Ziehen’s broadening of the associationist framework by his theory of associative constellations does not encompass the situational autonomy of the responses created by a particular task.

Watt concludes from his observations that the associationist explanation should be rejected. With it, Watt repudiates the bundle theory of consciousness, claiming that a continuous consciousness is “the condition for the occurrence of more complex factors, the task being one of them” (Watt, 1905, p. 422; translation by the author). Yet, his focal point remained the notion of the task. Apart from his observations about the variance in the experimental responses, he makes an important observation: “As we have seen, before and after the stimulus word with previous preparation, there occurs a pause, either a pause of waiting for the stimulus word or of waiting for the searched or appearing imagination” (Watt, 1905, p. 430; translation by the author). He, then, claims that this pause is the empirical manifestation of the consciousness of the task: “a task, thus, is a state of consciousness that exists in order to determine a sensible series of reproductions, and that can only be indicated as this (series), even comes to consciousness only as it” (Watt, 1905).

Mayer and Orth (1901), as well as Marbe (1901), first described a specific state of mind that was not identical with either volition or imagination:

“Experimental subject Mayer made the observation of an unspecific conscious process after hearing the stimulus word ‘poetic meter’ that was followed by the spoken word ‘trochee.’ In other cases, the subject succeeded to further characterize this experience. Orth observed that the stimulus word ‘mustard’ evoked such a peculiar conscious process which he meant to characterize as a ‘memory of a common figure of speech.’ It was followed by the reaction ‘seed.’ In all these cases, the subjects could not notice the presence of imaginations in the conscious state by which they specified the psychological phenomenon. We subsume all these manifold processes despite their obviously entirely different qualities under the name of ‘states of consciousness’ [*Bewusstseinslagen*]” (Mayer and Orth, 1901, p. 6; translation by the author).

This particular observation of *Bewusstseinslagen* triggered a fundamental debate about the role of intuition (in the sense of sensational, imaginative content) in thinking. In psychology of thought this crucial debate found its climax in the works of Bühler (1907, 1908a, 1908b). However, the notion of *Bewusstseinslage* is also pertinent for the question of what is the task since Watt claimed that the pause before and after the stimulus can be identified with these “states of consciousness.” In other words, the consciousness of the task cannot be identified with a simple imagination or volition, it is a particular state of mind. A state of mind that is an example of the specific form of experience that turned out to be the main topic for *Würzburgian* psychology of thought. However, just like Marbe, Watt did not succeed in characterizing the phenomenon further. The breakthrough for the understanding of the phenomenon of *Bewusstseinslage* was Bühler’s work that drew on Husserlian phenomenology.

### Narziß Ach’s Conception of the Task

The essential result from Watt’s investigations was the rediscovery of the task as a psychological phenomenon and its integration into the understanding of conscious life, implicitly refuting all externalist conceptions. The immanent psychology of the task could bridge the gap between instruction and actual goal-directedness. However, due to the associationist heritage of his idea and investigation Watt could not reach any further. One limitation of his approach was the imposed separation of the task from volitions as distinct states of consciousness. In contrast, Ach explicitly directed his research interest at the investigation of the will. This might seem unexpected for anyone who takes “psychology of thought” to be an exclusively intellectualist endeavor in the antiquated sense of a separation between faculties of the soul. Regardless, the *Würzburgian* psychology of thought never reduced the scope of its research merely to cognitive processes.

Nevertheless, like Watt, Ach’s starting point was influenced by associationist methodology. Continuing the work of his teacher Müller from Göttingen, he planned to complement the principles of connections between imaginations rather than raising doubts about the fundamental idea of connections between insulated mental elements. His investigations led him to the assumption of three principles, the first two are identical with Müller’s associationism: the associative and the preserving tendency. The third, Ach’s discovery, were the determining tendencies: “Determining tendencies can be understood as the effects that result from the particular imaginative content in the imagination of the goal and entail a determination in the sense of the meaning of this content of imagination” (Ach, 1905, p. 187; translation by the author).

The Brentanoesque distinction between an act and its content is the key to the idea of determining tendencies. Ach distinguishes the non-imaginative act of determination from the imaginative contents of consciousness. In order to tackle the prior, he coins the notion “being-conscious” (*Bewußtheit*) that has also been translated as “consciousness of objects” (Meyer, 1924) or “awareness” (Mason, 1913) and should not be confused with consciousness (*Bewußtsein*):

“With the help of the method of systematic experimental self-observation, we have obtained results for the analysis of the content of consciousness that repeatedly showed that a complex content of knowledge was present simultaneously. Withal, this knowledge was given without intuition, i.e., it did not contain phenomenological aspects, such as visual, acoustic, kinesthetic impressions or memory pictures of such impressions that would qualitatively determine the content that is given as knowledge. Such results occurred for all subjects who attended the systematic experimental self-observation. We shall call this being-present of a non-intuitional knowledge ‘being-conscious’ [Bewußtheit]” (Ach, 1905, p. 210; translation by the author).

The specific act of “being-conscious” is directed at non-intuitional content and the determining tendencies are “knowledge,” which is one of these contents. Despite this act-psychological distinction, Ach’s explanation remains teleological and the motif of determination is conceived as a strict mechanism: “being-conscious is the stronger, the greater the level of arousal of the imaginations that are at disposition, the stronger the arousal of the tendencies of reproduction” (Ach, 1905, p. 218; translation by the author). Consequently, Ach conceptualizes the determining tendencies as actual causal determination, ultimately forfeiting the independence from one-by-one associations that had been discovered by Watt – maybe because little to no attention had been paid to the autonomy of the act.

### Otto Selz’ Investigation of the Task

Ach had stated that “the effect of the determining tendencies does not only originate from present intentions but these tendencies can also be brought about by suggestive influence, commando, or by the task” (Ach, 1905, p. 196; translation by the author). Thereby, continuing Watt’s approach of integrating the notion of the task into an overarching unity of experience that does not depend on the internal-external dichotomy. Despite making progress in the analysis of the content of experience, his mechanistic teleology falls short on a phenomenological consideration of complexity on the side of the act. A further step was taken by Selz.

Selz explicitly criticizes both Watt and Ach for not having emancipated themselves sufficiently from the associationist traditions. Nevertheless, his work does not dismiss their line of investigation since he adopts the question: “What are the laws by which the determining tendencies cause the orderly course of the intellectual processes?” (Selz, 1913, p. 3; translation by the author). His answer, however, overcomes some of the limitations of his predecessors. In the center of his attention was the notion of the task, making him, among the *Würzburgian* psychologists of thought, the most elaborate commentator on the topic.

Selz’ first progression is empirical. Watt and Ach had investigated the variance of behavior under the condition of different tasks, separating different experimental groups by their respective tasks. Historically speaking, this step was necessary because it is the first occurrence of the phenomenon and, therefore, he could not manipulate it. Selz, however, determined the separation of groups is a weak point since the individual preparation cannot be investigated. Instead, he decided to alternate the tasks with every trial so that “at least the majority of the subjects has to find the solution instead of reproducing a solution that is already prepared” (Selz, 1913, p. 10; translation by the author). Consequently, he could examine cognitive activity when a subject is simulataneously faced with the stimulus and the task.

Watt and Ach had taken the task for a singular element of cognition that, despite its determining tendencies, could be identified with the moment of instruction. In contrast, Selz saw that the stimulus itself, being the material content of the cognitive act, partakes in the formation of experience and is not exchangeable and not a passive subject of association. His basic example is the verbal association of a name: When somebody is searching for the name of a popular person but cannot recall it immediately, another person might give them a hint by spelling the first letters of the name in question. These letters themselves already imply, for example, that the required response is verbal, a meaningful expression in line with certain rules of orthography or traditions of denotation.

Selz, then, makes a step of abstraction. He claims that these basic qualities are present even when the stimulus is very rudimentary, for example, when the subject only hears incomprehensible mumbling that, nonetheless, indicates the application of language. Thus, he concludes that a scheme of the reaction is present already in the stimulus material. The availability of this scheme, however, does not determine a factual response without the corresponding task. If the stimulus is presented without a task, the conscious state of the subject can be characterized as a “blank form” (see Selz, 1913, p. 218), a metaphor Selz uses to illustrate the nature of the scheme.

Likewise, a task without a stimulus leaves the subject in a comparable, but not identical, state of a “blank form”: If there is just a stimulus, the subject is prone to lose attention. If there is just a task, the subject will probably experience a tensed orientation toward the pending stimulus. Selz conceptualizes this orientation, which is induced by the consciousness of the task, as goal-orientation. Only the interaction of the task and the stimulus fulfils the determining tendencies, namely it determines the subject. Selz calls this interaction the “total task” (*Gesamtaufgabe*). The emphasis on the totality reflects the way in which he transgresses the associationist idea of the task as an element in a constellation. Instead of a constellation, Selz speaks of a complex.

A complex is a totality that resembles the idea of gestalt. It is a form that does not reside in any of its parts alone but in their entirety. Thus, a subject who perceives a vacancy within a complex may apprehend the task is to complete the complex. Selz describes this apprehension as the scheme that establishes the field for a response. Therefore, the “total task” is contrived to be the schematic unity of the stimulus and the “task in a broader sense” (with the “broader sense,” Selz refers to “the instructions from the examiner the subject must follow”; Selz, 1913, p. 178; translation by the author).

Notwithstanding this, Selz understands the determination of the responsive mechanism as the schematic relation of “complex association.” Thus, he does not forfeit associationism on the level of causation. When a stimulus and a task are given in the “total task,” the subject must react in a deterministic manner of “knowledge actualization,” filling the gaps of the complex. In other words, just as Watt and Ach, Selz remained faithful to the teleological explanations of reproductive thinking in the tradition of associationist psychology. Accordingly, it is safe to say that Selz, despite having critically expanded the notion of the task, did not surprise with his explanation of thought itself. His most valuable psychological legacy is the distinction between the instruction and the “total task,” not his mechanistic idea of reproductive and productive thought.

### The Task of Problem-Solving

Considering the problem of the task, the crucial question is whether 20th century psychology managed to conserve or even advance the level of reflection reached by the early psychology of thought. Without judging the value of the consecutive work in the field, it must be acknowledged that investigation of thought has undertaken a fundamental transformation, straying from the original discourse. The appeal of cybernetic cognitive sciences overrode the more sedate reflections on the nature of thought. The pioneers of modern-day problem-solving research, Newell and Simon, may have taken Selz into consideration, especially via the lecture of de Groot (Simon, 1999), but they did not reach his conceptual depth (Mack, 1997; van Strien and Faas, 2005). Lacking knowledge about the underlying controversies in the *Würzburgian* psychology of thought, they could not grasp its nuances. Instead, they salvaged the available material for their own interests in problem-solving. Ironically, in the process, they replicated some of the conceptual difficulties that had already been discussed by their predecessors.

Introducing imprecise terms like “task environment,” Newell and Simon whitewash the conceptual complexity of the underlying foundation: “The term task environment, as we shall use it, refers to an environment coupled with a goal, problem, or task – the one for which the motivation of the subject is assumed” (Newell and Simon, 1972, p. 55). They retreat to “constructed foundations” to try to bracket the motivational conditions of behavior that cannot be bracketed. They also conflate the notions goal, problem, and task.

This becomes increasingly clear in further passages, e.g., when they assume a “very simple problem situation where subjects can (and occasionally do) represent the task internally in quite different ways” (Newell and Simon, 1972, p. 63). Here, they return to an externalist concept of the task that ignores the critical progress made in psychology of thought bridging the internal-external dichotomy. Additionally, Newell and Simon even consider a task environment in the sense of a “Kantian Ding an sich” (Newell and Simon, 1972, p. 56). A task, thus, can be reduced to a “symbol structure” (Newell and Simon, 1972, p. 78) – returning to the associationist contention of a constellationist nature of thought. Furthermore, externalism is accompanied by representationalism:

“[W]e have insisted that we can know the objective task – ‘out there’ – only through its particular representations. There is no neutral way to describing the task environment. As a consequence, task instructions do much more than define the task; they provide, in addition, a specific representation of it that can serve to define an initial problem space, and even parts of an initial problem solving program for the subject” (Newell and Simon, 1972, p. 849).

For Newell and Simon, the representation of this external task environment coincides with the “goal” on the side of the subject as the internal information processing system^2^. Consequently, “[t]he task environment (plus the intelligence of the problem solver) determines to a large extent the behavior of the problem solver” (Newell and Simon, 1972, p. 788). This assertion is a clear setback compared with the earlier psychologists: Even the idea of determining tendencies had served to diversify the cognitive complexity instead of returning to a linear idea of causation. It should be seen as a mental function that is distinct from either associative or perseverant tendencies. The externalist conceptions of Newell and Simon, on the other hand, do not require more than associationist foundations.

In summary, it can be said that the more recent pioneers of psychological problem-solving research did not continue the line of investigation that was established by psychology of thought. The development of psychological research in the following decades, however, revealed that the progress of cybernetics and information sciences do not support psychological progress. Thus, a return to the conceptual depth of psychology of thought is necessary to advance the field.

#### Empirically Recovering the Complexity of the Task

The important achievements of psychology of thought lie beyond concept formation. The experimental investigations into thinking established a new format of introspective science, the method of “systematic experimental self-observation.” In the current discourse about first-person science of consciousness, this contribution to psychology is often underrated. Still, its relevance is more frequently recognized than the potential of phenomenologically revised problem-solving research (see Wendt, 2017). While introspection might have been the most important methodological topic for psychology of thought in the controversial delimitation from elementarist, functionalist, or behaviorist psychology, the discourse within the approach itself also considered more specific subjects, such as the nature of the task. With regard to present-day problem-solving research these latter considerations are more useful than a mere plea for a return to introspection. To put it another way, problem-solving research can harness the contribution of psychology of thought without the necessity of radical methodological concessions. This is the epistemological background of pseudo-interactivity.

Pseudo-interactivity tries to restore the discourse about the task as it has been undertaken in psychology of thought as a basis of problem-solving research. Its premise is the experimental observation of problem-solving and decision-making by the means of simulation that has been employed for the last decades in laboratory research with computers, especially in the context of so called “complex problem-solving” (Dörner and Funke, 2017). The basic experimental configuration is an imaginative scenario or a game – structurally comparable with the investigations on chess or cryptarithmetic by Newell and Simon. In its current form, this means that subjects are confronted with a digital “micro-world” (Funke, 1993) which simulates a more or less arbitrary content, such as the administration of a city (Dörner et al., 1983) or the scheduling of a daily routine (Holt et al., 2011).

Unlike traditional approaches that focus on algorithmic complexity, pseudo-interactivity reconsiders the meaning of the simulation in the light of the nature of the task itself. Drawing on Selz, the question is what schemes come to the fore when an experimental subject is asked to imagine they were, for example, the manager of a fictitious business. In the debate of the last decades, the difference between certain scenarios was a matter of formal difference, especially regarding the particular problem space. The paradigm of pseudo-interactivity, in contrast, shifts the attention from the possible operations of solving within a certain “micro-world” to the experiential conditions of the situation in which a subject partakes in a problem-solving task. Consequently, it is not decisive whether or not the subjects actually reach a possible solution or even improvement. Rather, pseudo-interactivity is designed to investigate the specifics of the experience, which allow a certain scenario to successfully simulate a problem.

## Method

Pseudo-interactivity is an experimental paradigm that encompasses behavioral studies, e.g., based on computer simulations. Its main purpose is the investigation of experiential differences between phenomenologically distinct types of situations, such as problems, challenges, and fatalities (see Wendt, 2017). Pseudo-interactivity allows for quantitative and qualitative measurements and it is not restrictive on the specific design. However, unlike comparable paradigms, it requires an explicit conceptual decision concerning the relationship between the material content, especially the task, and the experiential conditions of the experimental subjects.

The architecture of pseudo-interactive experiments combines two fields of psychology. First, it complements the field of complex problem-solving (CPS) research. CPS has worked with digital simulations in order to simulate so called complex problems. Funke uses five qualities to distinguish complex problems from simple problems: intransparency, dynamics, connectivity, polytely, and complexity (Funke, 2012). These qualities can be formalized and, thus, used as structural principles of a simulation. For example, a digital scenario is intransparent if the experimental subject cannot access all operating parameters that underlie the simulation as algorithms. Similarly, a simulation fulfils the quality of dynamics if the parameters, e.g., the arithmetic relation between inputs, change throughout the experiment. Therefore, the complexity of CPS can be effectively characterized as algorithmic complexity.

Algorithmic complexity, or, more specifically, algorithmically simulated complexity, is a term that is meant to describe a certain understanding of situations, such as the situation in a psychology laboratory, as complex systems. Dörner (2008) draws on the analogy of real-world complexity and a spring mattress in order to explain complexity: “everything is tied with everything else and nobody knows exactly how” (Dörner, 2008, 285; translation by the author). He continues describing complexity by some characteristics, such as “a great many variables”, “variables being ‘interconnected,’ or ‘weak’ causal relations.” What makes this understanding of complexity amenable to algorithmic implementation is the basic contention that complexity depends on the number, variety, and connectivity of “variables.” Dörner explicitly promotes that this form of complexity can be simulated by a machine, viz. a computer. The essential property of this kind of simulation is “the mathematical formulation of the hypotheses about the connections between the variables” (Dörner, 1996, p. 505; translation by the author). Algorithmic complexity, thus, is a mathematical representation of real-world inter-relatedness. Present-day CPS paradigms rely on this understanding of complexity as the criterion of validity for its simulations.

However, algorithmic complexity does not indicate the actual experience of complexity. The accumulation of a confusing number of ever-changing parameters does not guarantee that the experimental subject will actually experience a shift of attitude in comparison with, for example, playing chess. Accordingly, algorithmic complexity remains a label for a certain class of simulations that differ materially from so called simple problems, but not necessarily experientially. Certainly, the implicit motivation for the design of these simulations was the search for ecologically valid replications of real-life problems. However, the reality of problems does not (only) derive from their material multifariousness but from their vividness. The attempt to create more accurate and realistic simulations alone will not lead to more authentic experimental behavior.

In order to deal with this predicament, the second architectural principle is phenomenology. Unlike the search for greater ecological validity, phenomenology is not directed at the structural similarity between the simulation and an external situation. Instead, it tries to understand the essence of the experimental situation as a genuine experience in the lifeworld of an experimental subject. Hence, phenomenological psychology reinvigorates that inheritance from the psychology of thought.

The first reflective step of phenomenology has to be the critique of the CPS paradigm. What are the experiences that correspond with the respective moments of a simulation? The crucial insight is that no experimental subject actually encounters an intuitive problem when confronted with the cover story of a simulation, such as the administration of a business. Even in the fictitious case of an entirely ingenuous person who does not consider or question the experimental content, the best case will always be a projective imagination. Asking a person to imagine that they were the mayor of a city, as did Dörner and colleagues, can only result in imagination. This is the case if the instruction worked, that is, if it effectively manipulated the intention of the experimental subject, or if the subject willingly consented to follow the instruction (in the sense of a “hidden dialogue,” Lyons, 1970). Ultimately, however, this means that no simulation succeeds in presenting a factual scenario. The only immediacy a subject can experience is the laboratory situation – and therefore the constructed foundations of the simulation themselves. These foundations, however, are the reality of the task because of the nature and prerequisites of any laboratory situation. In other words, all the experimental content of problem-solving research is the result of a communicative influence or agreement to simulate.

Pseudo-interactivity does not try to resolve this constraint, but takes it as its point of departure. Instead of a more potent instruction or cover story, its design starts with the contention that the entire simulation relies on projection. Knowing that a person who engages with a CPS simulation can only imagine the situation they are supposed to be facing by projection, the need to construct an ecologically valid cover story is relieved. Instead, the decisions about the experimental design must concern the nature of the task. More precisely, in line with Selz, the composition of a pseudo-interactive experiment takes into consideration that all the content of the experiment forms the “total task.” Therefore, it becomes possible to design “total tasks” that express phenomenologically distinct modes of situations.

In order to manifest these situational modes (Wendt, 2017) in the experimental design, the core element of pseudo-interactive experimentation is semantic complexity instead of algorithmic complexity. While CPS experiments treat their parameters with a certain reluctance in order to cater to the common sense of the experimental subjects, pseudo-interactive experiments allow for extraordinary experimental behavior since it does not conservatively replicate the constraints of a plausible scenario. Only if there is a decisional margin may the experimental behavior represent the experiential diversity of simulated situations. In algorithmic complexity, there is only a margin for eventual solutions, not for the initial attitude.

Semantic complexity (viz. semantically simulated complexity), on the other hand, does not commit to the connectionist contention that complexity emerges from the fuzzy interaction of variables. However, it also does not reject it. Rather, it is a complementary conception of what happens in a complex situation. The fundamental idea that characterizes the semantically complex understanding of complex situations is “sense-making” and, more precisely in this context, “participatory sense-making” (De Jaegher and Di Paolo, 2007): “the process of generating and transforming meaning in the interplay between interacting individuals and the interaction process itself” (Fuchs and De Jaegher, 2009, p. 466). In a communicative situation, this intersubjective sense-making becomes its own realm of complexity– a complexity which can be simulated semantically.

In the context of CPS, the most important aspect of this specific form of complexity is that it cannot be reduced. “Sense” is manifested in meaningful actions that do not consist of elements that could be algorithmically represented as variables. As a consequence, psychological experiments that try to engage with this side of real-life complexity must employ different means. Semantically simulated complexity is one such means and tries to get a hold of “participatory sense-making” via the simulation of the semantic subtlety of communication. Pseudo-interactivity strives to cover this complexity in order to provide access to the nuances of experience.

How, then, can the appropriate experiential complexity be provoked? What tasks invite a variety of experiential attitudes? Pseudo-interactivity employs the simulation of personal interaction. Unlike practical decisions, such as the scheduling of daily routines, communicative interaction is genuinely manifold, and subjects may express themselves in a fictitious dialogue. Drawing on Fuchs, this form of fiction can be explained as “extended empathy”:

“To begin with, it entails an explicit, cognitive operation, namely, the conscious envisioning of the situation of the other, which often employs information about him that one could not infer directly from the situation at hand. Also, it involves an imaginative operation, that means, a transposition into an ‘as-if’ scenario (i.e., as if I were the other) which transcends the bodily or physical level. As a result, it seems necessary to differentiate between a primary, implicit, or bodily empathy and an expanded, explicit, or imaginative empathy. The latter already involves a certain degree of virtuality” (Fuchs, 2014, p. 158).

However, the operational advantage of CPS is that the experimental input is quantitative so that it can be used as a parameter for the algorithmic simulation and measured without transformation. However, it is not impossible to maintain these advantages without having to return to algorithmic complexity. One way would be to program a code that accommodates a free communicative input. While increasing the decisional margin, comparability is lost. Another way is to offer an ample variety of pre-coded verbal operators that offer a sufficiently wide margin of decisional alternatives and allows anticipation of the semantic complexity.

The following description and discussion of one of the first pseudo-interactive experiments employs this second design. In the given methodological context of this article, the main purpose of discussing this experiment is to illustrate the capabilities of pseudo-interactivity. The actual relevance of the paradigm for CPS, especially the theoretical background and the hypotheses that were investigated, will not be presented. It would require a lengthy explanation about the phenomenology of the problem (for a general outline see Wendt, 2017) in order to explain the precise meaning of the manipulations and measurements, distracting from the methodological purpose of the present discourse. The corresponding discussion about the experiment can be found elsewhere, alongside further experimentation (see Wendt, 2019).

### Exploratory Experimentation

A first pseudo-interactive experiment was conducted in early 2019 with a sample of 40 (34 female, 6 male, age *m* = 27, 3, *s* = 11, 4) students from Heidelberg University. Its premise was the investigation of the difference between an urgent and solvable situation (“problem”) and an urgent but unsolvable situation (“fatality”). In order to create continuity with CPS simulations, it was based on a variety of well-known experimental settings (“classical problems”), such as the “cannibals and missionaries” game^3^ (see Supplementary Figure S2), that were transformed to fit into the global setting of the simulation. It was implemented with the coding language MATLAB, version 2018a, and the integrated toolbox “psychtoolbox.”

The global setting was a traveling scenario. Participants were asked to imagine that they were traveling in a Spanish speaking country in South America and were to meet their friend at the train station within the next half an hour. Throughout the experiment, they could navigate the representation of their position on a map of the city Maracaibo in Venezuela (see Supplementary Figure S1). On their course, they would encounter up to four of the “classical problems” mentioned above. However, these “classical problems” were not presented by an instruction but as an encounter with a simulated person. The (pseudo-) interaction with this person could be executed by the application of 100 pre-coded operations, such as “concentrate oneself” or “provoke somebody”.

Most importantly, no action was demanded by an instructive task, neither in the beginning of the experiment nor in the case of an encounter within the simulation. Also, no action was required to finish the experiment. After 30 min, the simulation was terminated automatically, and the participants were asked to answer conclusive questions about their experience. Since the experimental design was inherently open, a variety of inputs were measured, such as the course of the participants on the maps and the actions in the “classical problems.” The most important measure, however, was the selection from the 100 operators (for a detailed discussion of the method see Wendt, 2019).

The difference between the two conditions was that the “problem” condition could solve the “classical problems” while the “fatality” condition could not. This difference was implemented by respective communicative responses by the simulated persons who accompanied the “classical problems.” For example, an elderly man who represented the “classical problem” of the “tower of Hanoi” asked the participant in a written message to help him carry a fragile machine. In the “problem” condition, it was possible to solve the task in the same fashion as one would solve the “tower of Hanoi.” In the “fatality” condition, the elderly man would interrupt the process after some actions and return all the parts of the machine back to their initial location. Hence, the difference did not exist on the logical level, i.e., the algorithmic architecture of variables, but on the operational and communicative level, i.e., the semantic material that is available for the participants’ sense-making.

In both conditions, the participants could find identical solutions to the “classical problems” by themselves. The “fatality” group of participants, however, could not implement their solution into the simulation. Based on phenomenological considerations about the problem (see Wendt, 2019), it was expected to find preferences for certain operators depending on the experimental group. Instead of discussing the hypotheses in detail, it is of greater importance at this point to revisit the methodological meaning of pseudo-interactivity.

While most research on problem-solving behavior presupposes that the task can be understood as a definite element of the experimental process, pseudo-interactivity investigates the development of the “total task” in a simulation with a wide decisional margin. Whether or not some simulation will be experienced as a problem in the emphatic sense of the word, cannot be guaranteed by instructions. Nonetheless, the interactions with the simulations are expressions of the sense making that occurs throughout experimentation and they express the particular attitude of the participants, the mode of their situation. Thus, it is necessary to include indicators of these attitudes into experimentation on CPS. Moreover, the method allows one to investigate the situational modes of experience themselves.

Taken together, the pseudo-interactive experiment was designed to make the original sense making of subjects who are confronted with a simulation accessible for psychological research, i.e., observable and measurable, instead of assuming “constructed foundations.” A step-by-step comparison of the pseudo-interactive and a typical CPS design may help to highlight the critical differences:

#### Introductory Phase

The purpose of the introduction in CPS used to be the unambiguous instruction. Briefing the participants is important for comparability. In contrast, in pseudo-interactivity the Selzian contention that the “total task” develops in confrontation with the stimulus material is taken into consideration. Therefore, the introduction does not give an unambiguous instruction although it remains clear that any content will already evoke a “blanket form” for eventual behavior. However, the purpose is to maintain the process of instruction incomplete.

#### The General Situation

The validity of CPS research depends entirely on the compliance of the participants. If a participant succeeds to solve a problem by chance or with, for example, a playful attitude, the results will be misguiding. Pseudo-interactivity does not depend on compliance but describes it in a phenomenologically refined form. Because of the decisional margin, the creativity of the participants can burgeon. If a participant disregards the introductory narrative and wants to test the experimental coding by the execution of mischievous patterns of behavior, they may do so, and it will reflect in their data. The purpose of the entire examination is to investigate under what circumstances certain forms of “total task” will be experienced.

#### Problem-Solving Phase

The research on CPS concentrates on the manipulation of parameters that can be judged as either favorable or unfavorable. Consequently, an optimization algorithm can standardize any CPS simulation. The operators in pseudo-interactivity, on the other hand, are not subject to a metric norm. If a person chooses certain communication actions to progress over others, they cannot be optimized in the mathematical sense of the word. However, from the point of view of coding, it is not a mistake to underpin the semantic complexity with an algorithmic architecture so that the responses of the simulation are strictly deterministic and thereby guarantee diagnostic objectivity and statistical comparability. The entire meaning of the actions results from the “pseudo-interaction,” i.e., the imaginative and projective cognitions of the participants.

#### Measurements

Complex problem-solving research normally investigates the input of integers that can be used as metrics for the statistical interpretation. Semantic complexity in its present operationalization does not have an obvious statistical measurement apart from the number of selected operators. Yet, given a theory of situational behavior, generalizations are possible. In the given experiment, a phenomenological theory of the problem, called “structure of problematic situations” (see Wendt, 2019), was used to evaluate the selection of operators. It includes five dimensions that distinguish different modes of situations, such as problems, challenges, and fatalities. These dimensions were “serious vs. playful,” “burdensome vs. comfortable,” “exploratory vs. committed,” “objective vs. subjective,” and “active vs. passive.” An independent rating of the 100 operators that were used in the experiment resulted in a distribution of representativity for each operator on the five dimensions. As a consequence, each selection of an operator in the simulation could be interpreted as an approximative expression on the respective situational dimension, if it had been rated accordingly.

#### Data-Model

The underlying data-model is the result of the two-step validation of the 100 operators (see above under “Measurements”). In the first step, the five behavioral dimensions that had been derived from phenomenological reflections, were used to sort the 100 operators. In the second step, this sorting was validated by expert rating. As a result, the operators that deviated by 1 to 2 standard deviations from the average score of the dimension, were given a score of 1, and the operators that deviated by more than 2 standard deviations from the average score were given a score of 2. This generalization of the ratings was meant to compensate for outliers in the ratings, increasing reliability. Consequently, the selection of each operator during the experiment can be measured on the respective dimensions with a score from −2 to 2 units (the meaning and scale of this unit derives from the standard deviation of the expert ratings). The resulting scores can be interpreted in isolation as a single event or in partial or complete aggregation. For example, a participant might have a cumulative score of 5 on the dimension “exploratory vs. committed” over the course of the entire experiment, meaning that she or he selected more operators that were rated as exploratory than those that were rated as committed.

#### Introspective Reports

A legacy of psychology of thought is that modern problem-solving research has employed the controversial method of “think-aloud-protocols.” The purpose of the data is to determine the detailed process structure of problem-solving, such as typical reactions to difficulties. The introspective data from pseudo-interactive experimentation, however, do not serve an equally specific purpose. Instead of the path of solution, the research is directed at the process of goal setting or problem finding. Hence, the pseudo-interactive manipulation serves the purpose of finding discrete modes of situations, such as distinct forms of goal setting and problem finding; a proviso is that the differences between the experimental conditions are as small as possible.

## Results

Due to the methodological focus of the investigation, reporting the results is primarily supposed to illustrate the conceptual significance of pseudo-interactivity. An interpretation of these tentative results as a contribution to the concept formation of problem-solving and problem finding has been published elsewhere (Wendt, 2019).

**FIGURE 1:**
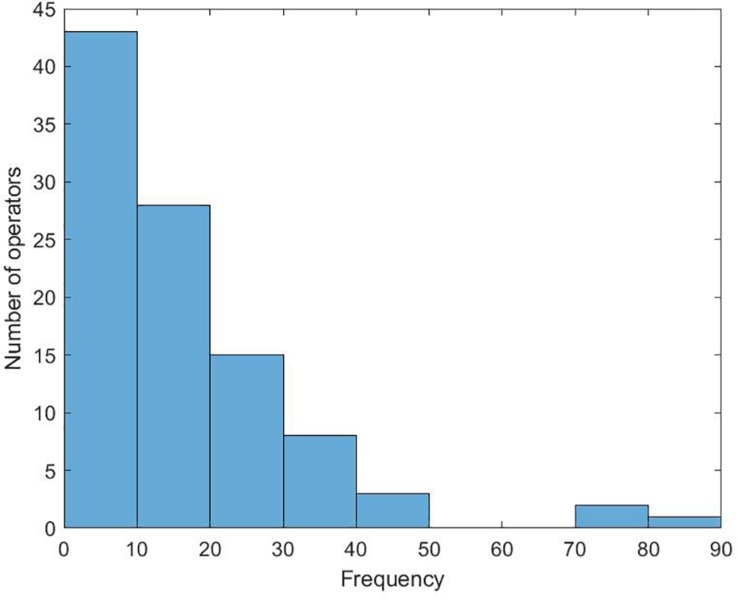
Histogram of the number of operators by frequency.

Of primary interest is a descriptive account of the situational difference between the participants that were faced with a “fatality” and those who could solve the “classical problems” that they were encountering. All 40 participants voluntarily used 1581 operators (there was no obligation to use them). Curiously, the distribution of operators between the two experimental groups was almost even (788 in the “problem” group and 793 in the “fatality” group). Overall, some operators enjoyed greater popularity than others, as can be seen in Figure 1. The most frequent operators were “ask somebody for help” (82 times), “question somebody” (75), and “understand the circumstances” (73). The least frequent operators were “strictly judge somebody” (1), “deny something” (2), “provoke somebody” (2).

These results indicate that the selection was not entirely random but guided by semantics. This is not trivial since the algorithmic function of the operators did not differ. In other words, on the side of the simulation, there was no difference between “asking” or “provoking” somebody. Yet, from the participants’ point of view, it was not easy to understand that the operators were redundant since the underlying mechanism was opaque and the reactions of the program did not repeat.

The differences between the two experimental groups can be described by the specific operator usage that did vary between them. For example, the group faced with “fatality” preferred the operators “evade the situation” (5 to 2), “limit oneself” (6 to 1), or “avoid something” (6 to 2). However, due to the low base frequency, these differences do not bear great explanatory weight. The opposite case shows a clearer pattern. The group of participants who could solve the “classical problems” showed prominent preference for operators, such as “reflect all circumstances” (17 to 8), “investigate something” (12 to 6), “question somebody” (43 to 32), “research into something” (16 to 7), or “take initiative” (25 to 16).

**FIGURE 2:**
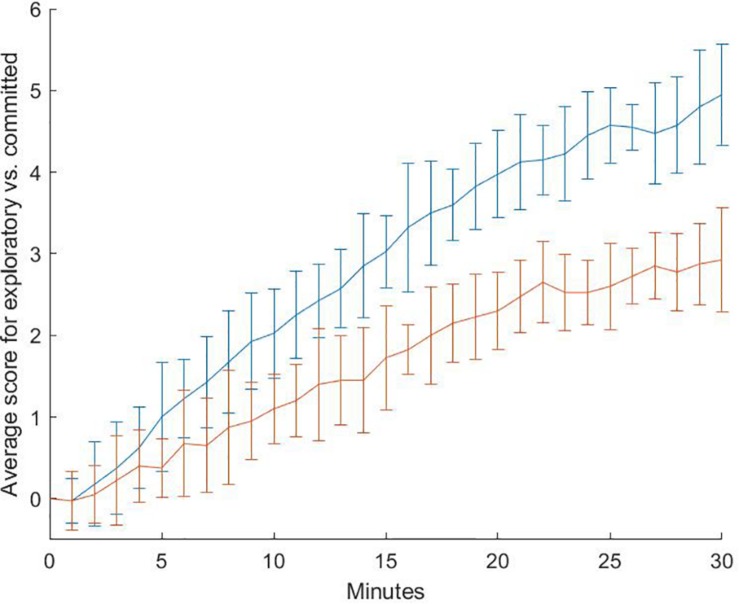
Cumulated average score on the dimension “exploratory vs. committed” for the duration of the experiment. A positive score represents “exploratory” operator selection, a negative score represents “committed” operator selection. The error bars show the standard deviation. Blue: “problem” condition (“classical problems” can be solved), red: “fatality” condition (“classical problems” cannot be solved).

Whilst not being the only operators with ostensible differences between the groups, these operators are of special interest because they belong to the group of operators that were considered salient to the dimension of “exploratory vs. Committed.” Accordingly, the overall change in the pattern of this dimension, which reflects operator preferences that relate to exploratory attitudes and behavior, helps to understand the situational difference between the two experimental groups. The general change can be illustrated by a line chart that represents the change over the course of the 30-min experiment (Figure 2).

The diagram shows the development of the average score on the dimension “exploratory vs. committed” by groups over the course of the 30-min experiment as a compound score of the rating for all selected operators. Generally, the preference of both groups tends toward operators that have been rated exploratory rather than committed. Yet, the participants of the “fatality” condition show a less pronounced tendency toward this extreme. On average, the operators which they applied during the entire experiment had an average score of about 2 units while the participants of the “problem” condition had a cumulated score of 5 units (for the exact procedure of acquisition for the unit of measurement see “Data-model” above).

**FIGURE 3:**
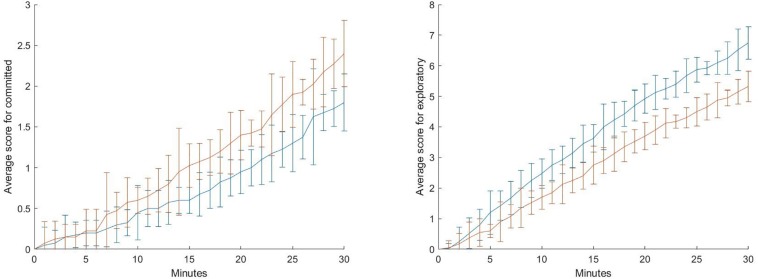
Cumulated average score for “committed” **(left)** and “exploratory” **(right)** for the duration of the experiment. The error bars show the standard deviation. Blue: “problem” condition (“classical problems” can be solved), red: “fatality” condition (“classical problems” cannot be solved).

The diagram shows that there is almost no difference for the operator preferences in the first minutes. This is consistent with the manipulation because the first difference in the encounters with the simulated persons who present the “classical problems” happens after 240s. From this point on, the two groups show a continuously growing gap of their average “exploration vs. commitment” score. For the total number of operators that were selected by the two experimental groups, there is a significant difference in the present sample on this dimension, *t*(38) = 1.93, *p* < 0.05 [medium effect size *d*_Cohen_ = 0.51 (0.19–0.83)]. However, this score is a composite of operator choices that score either on “exploration” or on “commitment.” A closer look can give a more fine-grained resolution of the decisional patterns (Figure 3).

These diagrams show that the difference between the two groups cannot be reduced to the participants in the “fatality” group either choosing less “exploratory” operators or choosing more “committed” operators. Actually, the composite score reflects a tendency for both extremes of the dimension. Consequently, the “fatality” condition can be described as an experimental situation in which participants have greater preference for committed operations, i.e., restricted and fixed actions, and lesser preference for exploratory operations, i.e., actions of discovery and experimentation, than participants of the “problem” condition^4^.

These quantitative aspects of the investigation are reflected in the introspective reports^5^. Participants in the “fatality” condition characteristically reported frustration and aggravation when asked about their general impression: “I tried to help and not achieving success was frustrating” (18 years old male); “I thought that I could help but everyone was unfriendly, so I kept moving” (23 years old female); “In some situations I felt desolated” (19 years old female). When asked about the experimental situation, several responses expressed reactance or even reluctance: “It lacked concrete instructions” (21 years old female); “There was no direction to the train station” (26 years old female); “I would have liked a better image of the city. In reality, one would see the surroundings and not a GPS” (20 years old female).

Participants in the “problem” conditions gave different responses. Despite having completed a simulation that was almost identical to the other experimental group, their reports reflect motivation and immersion: “I wanted to prove that I could move without fear in a foreign city” (22 years old female); “I had a good feeling after helping the merchant” (23 years old female); “I had the intention to help, so I felt useful” (23 years old female). Comments on the experimental situation did not express dissatisfaction but constructive criticism: “I would have liked to see some more things that were happening in the surrounding area” (49 years old female); “I liked that I actually had influence, for example, when calming the persons. I was not interested in music or the noise of the streets. Being able to choose between good and bad actions, was a good feature” (25 years old female), “I would have liked to play more and make more moves” (same person).

## Discussion

Clearly, differences between experimental groups concerning the apprehension of the general situation are a secondary effect that is inherent to all experimental designs. However, there are few approaches for the systematic investigation into the immanent structure of these differences and its experimental manipulation. Hence, exploratory results of pseudo-interactive research cannot be validated easily by empirical comparison. Some theoretical approaches have been provided by the discourse about the person-situation-dichotomy, for example, Pawlik (1978), or Lantermann (1980), or Frederiksen (1972). Yet, these postulations do not offer a method of validation but only models for the interaction between persons and objectified situations. A more pertinent form of validation is phenomenological psychology.

Unlike empirical concepts of the situation, such as the DIAMONDS-model (Rauthmann et al., 2014), phenomenology does not rely on the, by necessity, restricted range of empirical data. Rather, phenomenological psychology draws on eidetic reflection to grasp the nature of its subject matter. The contributions on the topic of the situation by, for example, the Utrecht school of phenomenology (Buytendijk, 1954; van den Berg, 1955; Linschoten, 1963), show that situatedness is an essential property of experience. Therefore, the empirical results made by applying pseudo-interactivity as an experimental paradigm cannot be seen as mere contingencies. On the contrary, they deal with a structure of experience that necessary for any further investigations of, for example, problem-solving. Only if the experiential characteristics of a certain experimental setup are examined, can interpretations about the subjective attitude toward the experimental content be justified.

In other words, research that relies on common sense in the construction and validation of their experimental designs bear the risk of uncontrolled mistakes about the actual situation they are creating. Pseudo-interactive investigations are a way to cope with this risk without having to rely on purely reflective considerations about the nature of the situation. The decisive step is to introduce a situational alternative that allows a comparison. Consequently, pseudo-interactivity allows psychology to move beyond the “constructed foundations” of experimental sciences. The peculiarities of the general situation in a laboratory, which can be abstractly described by notions like “compliance,” “demand characteristics,” or “social desirability,” can be described and compared in a concrete and phenomenologically adequate fashion (for a detailed discussion see Wendt, 2018).

The value of these descriptions and comparisons, however, depends on a return to the psychology of the task brought about by the *Würzburgian* psychology of thought. Watt’s fundamental insight was that tasks are not external to consciousness. If modern problem-solving research wants to be faithful to his conclusions, no formal criterion for experimental designs can be established that would guarantee that the experimental subjects conceive the laboratory situation as a task. The obvious backdoor of psychological interpretation is to assume that all recorded data conforms to the salient behavior. Yet, this division of behavior based on measurement must be arbitrary. Moreover, it constrains the psychological observation to predicted reactions. It disregards the creative responsibility of science.

Despite having touched on the phenomenon, Watt did not systematically investigate its relation to cognition. This step was taken by Ach. He claimed that the consciousness of a task could be explained as a determining tendency. For present-day psychology, this means that it is not enough to assume a single relative principle for all cognitive functions. Unlike tendencies of association, determining tendencies are characterized by anticipation. Thus, the emergence of task-consciousness should not be mistaken for a linear causation. Rather, it requires self-referential relations and thus subjectivity in the phenomenological sense of the word (for an understanding of circular causation see Fuchs, 2017).

Ach’s explanation still maintained an analogy between the associative, perseverant and determining tendencies. It was Selz who emphasized the border between associationist psychology and psychology of thought by claiming that the task was not a simple state of consciousness but a whole that should not be understood as a constellation but as a complex. Subsequently, the actual cognitive mechanism that may explain determining tendencies is a schematic actualization of knowledge. Problem-solving research might learn from this step that the situation created in an experimental setup cannot be predictably modified by changing singular elements. The experience that an experimental subject will have when confronted with a laboratory situation will necessarily be complex and difficult to predict. Thus, a rigorous empirical approach to investigate these complex subjective dynamics is required since common sense assumptions are neither reliable nor controllable. Pseudo-interactivity helps bridge the conceptual gap. It tries to make the subjective experiences of the experimental subjects traceable, or, to borrow a term from ethnomethodology, “accountable.”

The presented exploratory experiment that tries to distinguish the experience of a “fatal” from a “problematic” situation, demonstrates a certain resemblance between pseudo-interactivity and ethnomethodology. Ethnomethodology tries to discover the exact process of creating rules that give structure to everyday life. These rules and norms are considered “methods” from the subject’s experiential point of view: “the most important assumption that drives ethnomethodological approaches is the methodic and orderly character of everyday activities that appear chaotic and messy at first glance” (Reeves et al., 2016, p. 330). However, ethnomethodology is restrained to a sociological perspective and shies from introspective reports and considerations about psychological processes.

Another like-minded project is the particular ecological psychology that has emerged from phenomenological psychology (e.g., Graumann and Kruse, 1998). It discusses the notion of the situation in a holistic fashion, but it is emancipated from the rather individualistic thought psychology in order to embrace social psychology. Still, its contributions can help to understand better the complexity of the situation as a meaningful and complex aspect of life. Likewise, anthropological psychology (e.g., von Uslar, 1973) describes the existential conditions of situatedness. Nevertheless, none of these approaches has developed an experimental nexus to current problem-solving research that carries the conceptual heritage of psychology of thought. Pseudo-interactivity may fill this gap. However, the experimental designs have to be improved and validated.

The present investigation demonstrated the utility of pseudo-interactivity. It foreshadowed the major challenges for the paradigm: the experimental design requires a degree of precision and structure that could not yet be reached. For example, the success rate of the “classical problems” is at 17.9%. Thus, the fatality by design due to practically unsolvable encounters might be conflated with apparent insolubleness by difficulty. The future development of comparable experiments will shed light on the practicability and the scope of the paradigm.

## Data Availability Statement

The datasets generated for this study are available on request to the corresponding author.

## Ethics Statement

Ethical review and approval was not required for the study on human participants in accordance with the local legislation and institutional requirements. The patients/participants provided their written informed consent to participate in this study.

## Author Contributions

The author confirms being the sole contributor of this work and has approved it for publication.

## Conflict of Interest

The author declares that the research was conducted in the absence of any commercial or financial relationships that could be construed as a potential conflict of interest.

## Footnotes

1 Going farther than Watt, August Messer concluded that the idea of a purely free association is unrealistic: “In the first experiment, the task was given to associate the first arbitrary word that comes to mind. However, it can be observed most frequently that the subjects were searching for a word that stood in a meaningful relation to the stimulus word, even without being aware of it. Thus, they subconsciously set themselves a more specific task” (Messer, 1906, p. 22; translation by the author).

2 This idea, however, does not neglect much of the previous discourse because Watt, Ach, and Selz did not overcome their teleological convictions, either. This lack of critical flexibility reveals an important difference between the rather cognitivist group of *Würzburgian* psychologists of thought and a phenomenological alternative, such as Messer (1908, 1911), Lindworsky (1916), and, later, Graumann (1955, 1960).

3 Logic transformations in the setting of three missionaries who must escort three cannibals over a river. They may use a boat for two persons. It is prohibited that there are more cannibals than missionaries on either of the sides of the river at any point in time.

4 These results are in accordance with the hypotheses posed by the structure of problematic situations (Wendt, 2019). These hypotheses and their rationalization do not have to be discussed on occasion of these primarily methodological considerations.

5 All translations by the author.
