# Supplementary figures and images for: The Problem of the Task. Pseudo-Interactivity as an Experimental Paradigm of Phenomenological Psychology
Source: Front Psychol. 2020 Apr 30;11:855. doi: 10.3389/fpsyg.2020.00855 (PMC7204526; doi:10.3389/fpsyg.2020.00855)

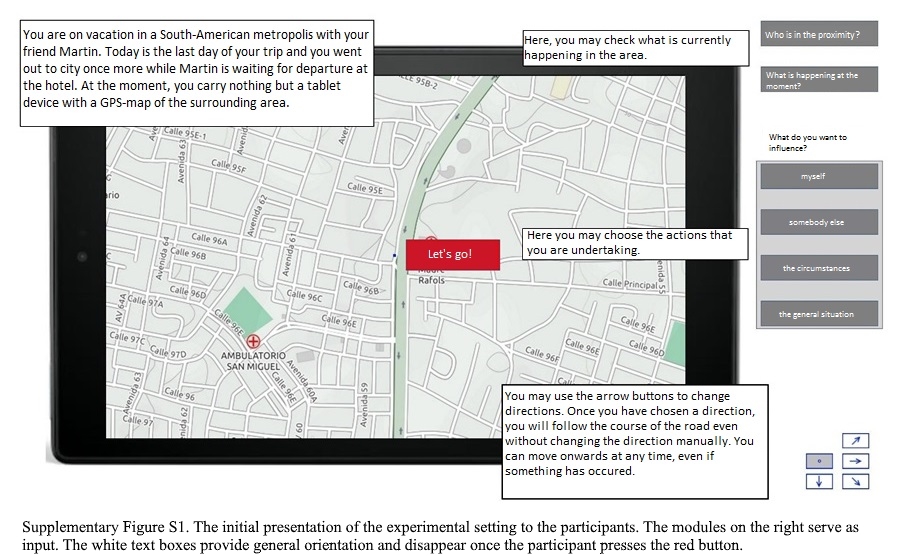

Supplement: Supplementary file 1 [file Image_1.jpg]

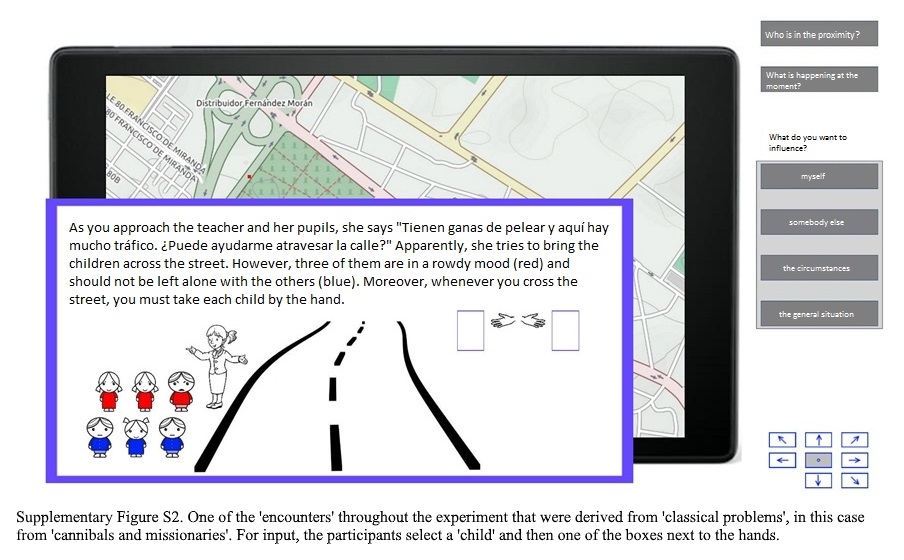

Supplement: Supplementary file 2 [file Image_2.jpg]
